# The transcription factor Leu3 shows differential binding behavior in response to changing leucine availability

**DOI:** 10.1093/femsle/fnaa107

**Published:** 2020-06-26

**Authors:** Christoph S Börlin, Jens Nielsen, Verena Siewers

**Affiliations:** Department of Biology and Biological Engineering, Chalmers University of Technology, Kemivägen 10, SE-41296 Gothenburg, Sweden; Novo Nordisk Foundation Center for Biosustainability, Chalmers University of Technology, Kemivägen 10, SE-41296 Gothenburg, Sweden; Department of Biology and Biological Engineering, Chalmers University of Technology, Kemivägen 10, SE-41296 Gothenburg, Sweden; Novo Nordisk Foundation Center for Biosustainability, Chalmers University of Technology, Kemivägen 10, SE-41296 Gothenburg, Sweden; BioInnovation Institute, Ole Maaløes Vej 3, DK2200 Copenhagen N, Denmark; Department of Biology and Biological Engineering, Chalmers University of Technology, Kemivägen 10, SE-41296 Gothenburg, Sweden; Novo Nordisk Foundation Center for Biosustainability, Chalmers University of Technology, Kemivägen 10, SE-41296 Gothenburg, Sweden

**Keywords:** Leu3, transcription factor, ChIP-seq, yeast

## Abstract

The main transcriptional regulator of leucine biosynthesis in the yeast *Saccharomyces cerevisiae* is the transcription factor Leu3. It has previously been reported that Leu3 always binds to its target genes, but requires activation to induce their expression. In a recent large-scale study of high-resolution transcription factor binding site identification, we showed that Leu3 has divergent binding sites in different cultivation conditions, thereby questioning the results of earlier studies. Here, we present a follow-up study using chromatin immunoprecipitation followed by sequencing (ChIP-seq) to investigate the influence of leucine supplementation on Leu3 binding activity and strength. With this new data set we are able to show that Leu3 exhibits changes in binding activity in response to changing levels of leucine availability.

## Teaser

Leu3, the main regulator of leucine biosynthesis in yeast, shows reduced binding activity in the presence of branched chain amino acids.

## INTRODUCTION

In the yeast *Saccharomyces cerevisiae*, leucine biosynthesis is controlled on a transcriptional level mainly by the interplay of two transcription factors (TF), the general amino acid metabolism regulator Gcn4 and one specific for leucine, called Leu3 (Friden and Schimmel 1987; Kohlhaw 2003). As the three branched-chain amino acids, leucine, valine and isoleucine, share common genes in their biosynthetic pathways, Leu3 also influences valine and isoleucine biosynthesis activity (Kohlhaw 2003).

Leu3 is a TF belonging to the large class of zinc-knuckle type TFs, which often form homodimers and are depending on coordinating two zinc ions for binding to the DNA. An interesting feature of Leu3 is its ability to bind alpha-isopropylmalate, an intermediate of the leucine biosynthetic pathway. It has been shown that this binding leads to a conformational change and is necessary for its activation (Hahn and Young 2011). It has been reported that Leu3 binds to seven genes in the biosynthetic pathway of leucine starting from pyruvate, namely *ILV2*, *ILV3*, *ILV5*, *LEU1*, *LEU2*, *LEU4* and *BAT1* (Kohlhaw 2003; MacIsaac *et al*. 2006). The Saccharomyces Genome Database (Cherry *et al*. 2012) lists 140 known gene targets for Leu3, only 125 of them however are based on direct evidence from chromatin immunoprecipitation experiments in multiple *S. cerevisiae* strains and different conditions.

In earlier publications about Leu3, it has been reported to always bind to its targets, independent of the leucine availability and its activation status (see (Kirkpatrick and Schimmel 1995; Harbison *et al*. 2004). In a previous study from our lab, in which we used high resolution chromatin immunoprecipitation followed by exonuclease treatment and sequencing (ChIP-exo), we observed that Leu3 showed differential binding behavior at four different cultivation conditions, covering respiratory and fermentative growth (Holland *et al*. 2019). The four conditions were, however, so different that it was difficult to assess if this change in binding behavior was connected to changes in leucine availability or caused by other effects. Here, we present a follow-up study with a set of experiments where the only changing variable is the availability of the three branched-chain amino acids, leucine, valine and isoleucine.

## EXPERIMENTAL PROCEDURES

### Yeast strains and growth conditions

In this experiment, the *S. cerevisiae* strain CEN.PK 113–5D (Δ*ura*3 of CEN.PK113–7D (van Dijken *et al*. 2000)) with a 9xMyc tag at the C-terminal end of the Leu3 coding sequence and the *URA3* gene from *K. marxianus* was used as previously described (Holland *et al*. 2019).

For the experiments, the cells were first grown over night in shake flasks at 30°C and 200 rpm shaking in minimal medium with 2% glucose. The minimal medium consisted of 14.4 g/L KH_2_PO_4_, 0.5 g/L MgSO_4_, 7.5 g/L (NH_4_)_2_SO_4_, 1 mL/L trace metal stock solution, 1 mL/L vitamin stock solution. The pH of the media was adjusted to 6.2–6.3 using KOH pellets. Trace metal stock solution components (per liter of stock solution) were: 15.0 g EDTA-Na_2_, 4.5 g CaCl_2_·2H_2_O, 4.5 g ZnSO_4_·7H_2_O, 3 g FeSO_4_·7H_2_O, 1 g H_3_BO_3_, 0.84 g MnCl_2_·2H_2_O, 0.4 g Na_2_MoO_4_·2H_2_O, 0.3 g CuSO_4_·5H_2_O, 0.3 g CoCl_2_·6H_2_O and 0.1 g KI. Vitamin stock solution components (per liter of stock solution) were: 25 g myo-inositol, 1 g nicotinic acid, 1 g calcium pantothenate, 1 g pyridoxine HCl, 1 g thiamine HCl, 0.2 g 4-aminobenzoic acid and 0.05 g biotin.

The next day, 10 shake flasks were set up at a starting OD600 of 0.05 in 20 mL of minimal medium with 2 glucose feed beads (Kuhner Shaker, Herzogenrath, Germany, 12 mm high release glucose FeedBeads, Art. No. SMFB08001) at 30°C and 200 rpm shaking.

After 70 h, the cells were treated with one of the following: water as control, low level of leucine (final concentration 20 mg/L), high level of leucine (final concentration 100 mg/L), high level of isoleucine (final concentration 100 mg/L) or high level of valine (final concentration 100 mg/L). Each treatment was performed in duplicates. After an additional 2 h, 50 OD600 of cells were collected for chromatin immunoprecipitation followed by sequencing (ChIP-seq) and 5 OD600 of cells were collected for mRNA extraction and qPCR analysis.

### Chromatin immunoprecipitation

DNA–protein complexes were cross-linked for 15 min at room temperature in a 1% formaldehyde solution and the cross-liking reaction was quenched for 5 min at room temperature by addition of glycine to a final concentration of 125 mM. Finally, the cells were washed twice with TBS (10 mM Tris-HCl pH 7.5, 150 mM NaCl) and then snap frozen in liquid nitrogen.

The cells were thawed in ice-cold lysis buffer (50 mM HEPES/KOH pH 7.5, 140 mM NaCl, 1 mM EDTA, 1% Triton X-100, 0.1% Na-deoxycholate, 0.05% SDS and 0.2% Sigma P8215 protease inhibitor cocktail) and transferred to MP Biomedicals Lysing Matrix C glass bead. For cell disruption, the Precellys FastPrep was used with 6 cycles of 25 s and 7400 rpm. The cells were then transferred to a fresh 5 mL Eppendorf tube and more lysis buffer was added to a final volume of 3 mL. Using a Branson digital sonifier 250 with a 1/8-inch microtip, the cells were then sonicated for 5 min with pulses of 10 s on and 20 s off at an amplitude setting of 30%. After sonication, the solution was centrifuged at 21 000 *g* for 15 min at 4°C to remove the cell debris.

The immunoprecipitation was done using 15 µL of Pierce Anti-c-Myc magnetic beads over night at 4°C. After that the beads were washed with lysis buffer without protease inhibitor and SDS, then with IP wash buffer (10 mM Tris-HCl pH 7.5, 250 mM LiCl, 2 mM EDTA, 0.5% Triton X-100 and 0.5% Na-deoxycholate) and then with 10 mM Tris-HCl pH 8.0 plus 0.025% Tween20.

After washing, the DNA was eluted from the beads and the cross linking was reversed using Myc-elution buffer (25 mM Tris-HCl pH 8.0, 2 mM EDTA, 200 mM NaCl, 0.5% SDS) with 0.66 µg/µL protease K and 0.083 µg/µL RNase A. The DNA was extracted using the Promega ProNex size-selective purification system, using a double selection with first 1 × volume for an upper length cutoff of 1000 bp and then 2 × volume for a lower cutoff of 100 bp.

### Library preparation for Oxford Nanopore MinION sequencing

For sequencing, an Oxford Nanopore MinION sequencing device was used and the library preparation was done using the Oxford Nanopore PCR Barcoding Kit (SQK-PBK004). The purified and size selected DNA were first prepared for adapter ligation using the NEBNext Ultra II End Repair/dA Tailing Module and purified again using ProNex. The ligation of the Barcode Adapters (BCA) was done using the NEB Blunt/TA Ligase MasterMix for 15 min at 25°C. After another purification using ProNex, the DNA was amplified with the Barcode Primers (LBW 01–10) for 25 cycles using Phusion DNA polymerase. The assigned barcodes for each sample are shown in Table 1.

**Table 1.** Used Oxford Nanopore Barcodes for each sample.

Sample	# Barcode
Control 1	1
Control 2	3
20 mg/l Leucine 1	2
20 mg/l Leucine 2	4
100 mg/l Leucine 1	5
100 mg/l Leucine 2	6
100 mg/l Isoleucine 1	7
100 mg/l Isoleucine 2	8
100 mg/l Valine 1	9
100 mg/l Valine 2	10

After a final purification of the DNA using 1.75 × volume for a lower cutoff of 200 bp, the 10 samples were pooled in equal ratios to a total of 100 ng of DNA in 10 µL of 10 mM Tris-HCl pH 8.0 with 50 mM NaCl. Then the Rapid Adapters (RAP) were added and the Oxford Nanopore flow cell (version R9.4) was primed and loaded. Finally, the libraries were sequenced using the Oxford Nanopore MinKNOW software suite using two flow cells after each other to obtain a total of 5.1 million reads.

### Analysis of sequencing data

The raw fast5 sequence data files were basecalled using the standalone Oxford Nanopore basecaller Guppy (version 3.6.0) with a quality threshold of 7, direct quality filtering and direct demultiplexing and barcode trimming.

The obtained fastq reads were then mapped to the CEN.PK113–7D genome (Salazar *et al*. 2017) using Bowtie2 (Langmead and Salzberg 2012) with the local read map setting. The obtained sam files were then subsequently converted into bam files using samtools (Li *et al*. 2009). Leu3 binding peaks were detected using GEM version 3.4 (Guo, Mahony and Gifford 2012), which was also used to calculate the signal to noise ratios (SNR) for the peaks based on the combined replicates, as well as to estimate the local background noise (also performed by GEM). The ChIP-seq distribution file from GEM was used together with the following parameters: q-value threshold of 0.001, length of kmer between 5 and 18, smoothing width of 30 (default value), minimum number of events of 5 and a maximum read count per base position of 4 (default value). For each condition both replicate sequencing files were used as inputs for GEM, which uses an internal normalization approach to combine replicates and outputs an averaged final score. The resulting peaks where assigned to genes based on their distance to the transcription start site (TSS), obtained from a previous study (Börlin *et al*. 2019). Peaks with a distance less than 1000 bp away from a TSS annotation (independent of upstream of downstream) were assigned to the corresponding gene. If a peak was close enough to two genes, it was assigned to both genes.

### Measuring mRNA expression levels using qPCR

For mRNA extraction, the cells were disrupted using 500 mg of acid washed glass beads with a diameter of 425–600 µM on a Precellys FastPrep for 40 s with 7200 rpm. RNA was subsequently extracted using the RNeasy® Mini Kit from Qiagen, Hilden, Germany. RNA quality and quantity were assessed using a ThermoFischer, Waltham, MA, USA, NanoDrop and 2 µg of RNA were reverse transcribed into cDNA using the Qiagen QuantiTect Reverse transcription kit. Expression levels of *LEU1* were measured using the ThermoFisher Dynamo Color Flash SYBR Green qPCR kit on an Agilent Technologies, Santa Clara, CA, USA, Stratagene Mx3005p (2 step qPCR protocol, 10 min initialization at 95°C, 40 cycles of: 30 s 95°C, 60 s 60°C). For control of input cDNA amounts the levels of *LEU1* were normalized using the ΔΔCt method to measured levels of *TAF10* (3 step qPCR protocol, 10 min initialization at 95°C, 40 cycles of: 30 s 95°C, 60 s 56°C, 30 s 72°C). The used primers are shown in Table 2. For both qPCR measurements the samples were analyzed in technical duplicates.

**Table 2.** Used qPCR primers.

Primer	Sequence
LEU1 forward	ATCGTCCACACCATCGGTCCC
LEU1 reverse	GGCCAGCGAACCAAAGGCAC
TAF10 forward	ATATTCCAGGATCAGGTCTTCCGTAGC
TAF10 reverse	GTAGTCTTCTCATTCTGTTGATGTTGTTGTTG

## RESULTS AND DISCUSSION

**Figure 1.**
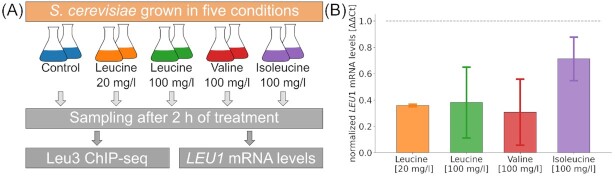
Experimental setup and validation of treatment effect. **(A)** Overview of experimental setup used. **(B)** Measured *LEU1* mRNA levels (normalized to *TAF10* and control levels using ΔΔCt method) for the four treatment conditions.

In this study about the condition dependent behavior of the leucine biosynthesis controlling transcription factor Leu3, we measured the binding activity of Leu3 in *S. cerevisiae* using chromatin immunoprecipitation followed by sequencing (ChIP-seq). The only variable changing in these experiments was the addition of leucine or the two other branched-chain amino acids, valine and isoleucine to the media. For that, the cells were treated with either: (i) water as control, (ii) 20 mg/mL leucine, (iii) 100 mg/L leucine, (iv) 100 mg/L valine or (v) 100 mg/L isoleucine. After the treatment cells were collected for ChIP-seq and mRNA extraction to validate that the treatment does affect expression of known binding targets of Leu3. The experimental setup is summarized in Fig. 1A.

Using qPCR measurements for *LEU1* mRNA levels, we observed that the chosen treatments affected the expression of *LEU1*, one of the major genes in the biosynthetic pathway for leucine. As shown in Fig. 1B, the addition of any of the three branched-chain amino acids lowered the expression of *LEU1*, with leucine and valine having the similar effect with a reduction to around 40% of the control values. This reduction of *LEU1* expression in the presence of leucine has also been reported previously (Hsu and Schimmel 1984). These changes in expression validate the treatment strategy allowing us to be confident that observed changes in Leu3 binding patterns are caused by the availability of leucine, valine or isoleucine.

**Figure 2.**
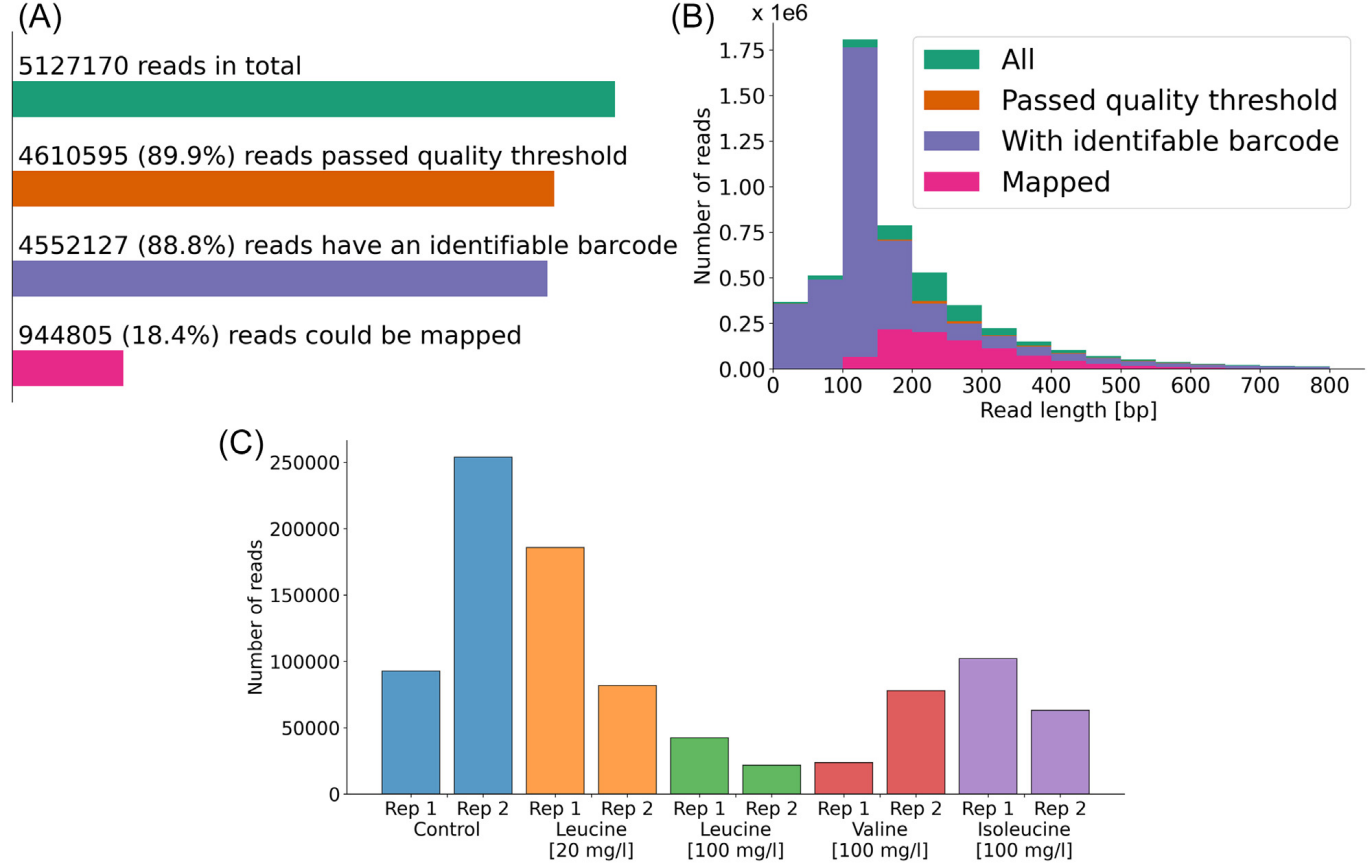
Overview of sequencing data. **(A)** Number of sequenced reads for all conditions combined and how many passed the different filtering steps. **(B)** Histogram of the read length of all reads across the conditions and change of the distribution after the different filtering steps. **(C)** Number of mapped reads per sample (each sample has its own unique barcode).

The obtained ChIP-seq raw sequencing data from Oxford Nanopore MinION were base called and demultiplexed and then mapped to the genome. In total, 5.12 million reads were sequenced, of which more than 88% passed the quality threshold and had an identifiable barcode sequence. Of these, 944 805 reads (18.4%) could be mapped to the reference genome. An overview of the respective number of reads is shown in Fig. 2A. The distribution of read lengths is displayed in Fig. 2B, with an average read length of 193. One can observe that there is no read length bias regarding reads that passed the quality threshold or had an identifiable barcode, but there is a strong bias for longer reads with regard to mapping to the reference genome. This is not surprising as mapping a read with sequencing errors to the genome is easier if the read is longer. Therefore, the average read length of mapped reads is 271. The number of mapped reads per sample also varies between 21k and 253k as shown in Fig. 2C.

**Figure 3.**
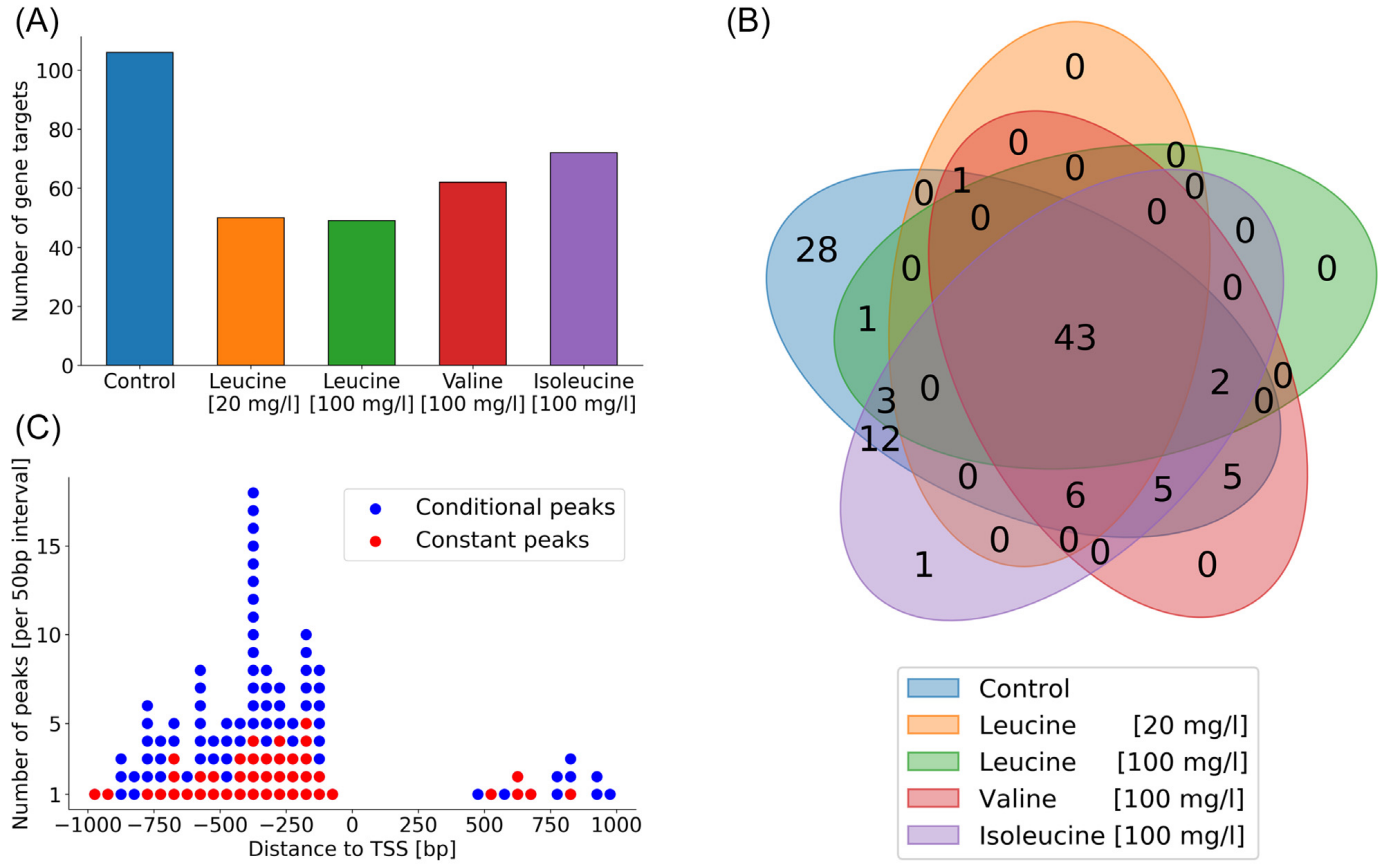
Detected gene targets for Leu3. **(A)** Number of detected Leu3 binding targets for each of the five conditions. Only peaks detected by GEM with a signal to noise ratio of ≥ 2 that are within 1000 bp of a TSS were taken into account. **(B)** Distribution and overlap of the Leu3 gene targets between the five conditions. Venn diagram made using the python package pyvenn, version 0.1.3, available from PyPI. **(C)** Distribution of peak distances to the transcription start site (TSS) binned in 50 bp intervals, divided into peaks detected in all five conditions (constant peaks) and peaks that were only detected in some conditions (conditional peaks).

Starting from the mapped reads, peaks in TF binding were detected using GEM (Guo, Mahony and Gifford 2012) and only peaks with a signal-to-noise ratio (SNR) of at least two were retained. The background noise levels were estimated using GEM. These peaks were assigned to genes based on the distance to the transcription start site (TSS). The number of gene targets in each condition is shown in Fig. 3A and the complete list of binding targets and the corresponding binding strengths can be found in Table S1 (Supporting Information). Interestingly, the control condition was the condition with the highest binding activity with 106 identified gene targets out of a total of 107. The only gene that is not bound in the control condition is SOK2 that is weakly bound with the addition of isoleucine. The addition of leucine led to a strong reduction in number of gene targets, with 50 gene targets in the low leucine and 49 in the high leucine condition. The addition of the two other branched-chain amino acids, valine and isoleucine, resulted in less reduction of binding (62 and 72 targets for valine and isoleucine respectively). As Leu3 is mainly a regulator of leucine biosynthesis and not a general branched-chain amino acid biosynthesis regulator, it is not surprising that valine and isoleucine showed less effect. In total 107 genes were bound in at least one of the conditions and their distribution and overlap across the five conditions is shown in Fig. 3B. Here, we could observe that less than half of all gene targets were bound at all five conditions. This binding behavior is in contrast to earlier literature (Kirkpatrick and Schimmel 1995) but confirms what we saw before in ChIP-exo data from different bioreactor cultivations (see (Holland *et al*. 2019)

We also investigated the spatial distribution of the detected peaks in relation to the transcription start site (TSS) of their target genes and the distribution is shown in Fig. 3C. The peaks were classified as either constant, meaning that they were bound in all five condition, or as conditional if they showed no binding in at least one condition. As expected, the majority of all peaks are 750–100 bp upstream of the TSS with no binding observed directly around the TSS. There is also no marked difference in the spatial distribution of the two types of detected peaks. Using the motif discovery software MEME (Bailey and Elkan 1994), we observed that there was no significant difference in the most common motif for peaks that are either classified as constant or conditional (data not shown).

**Figure 4.**
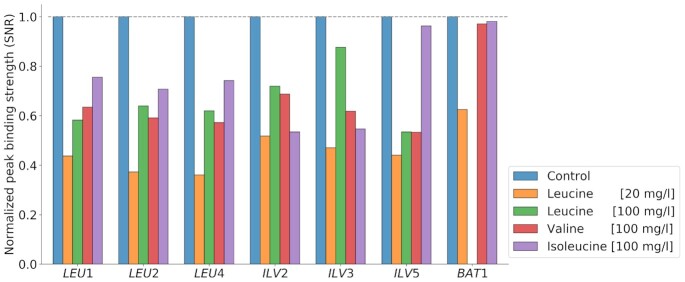
Leu3 binding strength on leucine metabolic genes. Peak binding strength (signal to noise ratio, SNR) normalized to control levels of Leu3 on genes involved in leucine biosynthesis in the five different conditions used.

In the list of 43 gene targets that showed binding in all conditions, most of the leucine biosynthetic genes, like *LEU1*, *LEU2*, *LEU4*, *ILV2*, *ILV3* and *ILV5*, are included. This could explain why previous studies have found that Leu3 always binds to its targets. The newer technologies used here however allows us not only to obtain a binary output of binding/no-binding but also to qualitatively assess the binding strength using the SNR of the binding. For the aforementioned genes this is displayed in Fig. 4, together with *BAT1*, another important gene in leucine biosynthesis, which was however not bound in the presence of high amounts of leucine. One can observe that the binding strength for all seven genes was reduced with the addition of any of the three branched-chain amino acids and that leucine had the strongest effect. On average, the addition of leucine to the media reduced the binding strength by more than 50%. Interestingly, the lower level of leucine seems to have a bigger effect with a reduction of 60%, than the high level, with a reduction of only 42%. The high level of leucine still had a stronger effect than valine and isoleucine, with a reduction of only 33% each. The overall reduction means that even though these genes are still bound in the presence of leucine the binding activity of Leu3 is reduced. This can lead to a reduction in gene expression levels, which is exactly what we can observe for the mRNA levels of *LEU1* (see Fig. 1B).

## CONCLUSION

Here, we could show that the binding activity of the leucine biosynthetic TF Leu3 is affected by changing environments and specifically by changes in the availability of leucine and the two other branched-chain amino acids, valine and isoleucine. This is in contrast to earlier molecular studies from the 1990s (Kirkpatrick and Schimmel 1995), but is in line with our published data using ChIP-exo in another set of four different conditions besides the five tested here (Holland *et al*. 2019).

## ACCESSION NUMBERS

The Nanopore sequencing data and results can be found under the ArrayExpress accession code E-MTAB-8820.

## Supplementary Material

fnaa107_Supplemental_File
